# The Effectiveness of Lifestyle Changes in Glycemic Control among Pregnant Women with Gestational Diabetes Mellitus

**DOI:** 10.3390/medicina59091587

**Published:** 2023-09-01

**Authors:** Duc Cuong Le, Thanh Binh Vu, Thi Nuong Tran, Thi Ly Nguyen, Thanh Binh Nguyen, Duy Cuong Nguyen, Van Thuan Hoang

**Affiliations:** Thai Binh University of Medicine and Pharmacy, Thai Binh 410000, Vietnam; cuongldvn@gmail.com (D.C.L.); binhvt@tbump.edu.vn (T.B.V.); nuongtrandr@gmail.com (T.N.T.); lythaibinh81@gmail.com (T.L.N.); binhnt@tbump.edu.vn (T.B.N.); cuongnd@tbump.edu.vn (D.C.N.)

**Keywords:** lifestyle changes, diet, physical exercise, gestational diabetes mellitus, glycemic control, diabetes

## Abstract

*Background and Objectives*: Gestational diabetes mellitus (GDM) is a type of diabetes that develops during pregnancy and affects approximately 10% of pregnant women worldwide. Understanding the impact of lifestyle changes on glycemic control in GDM is important for improving maternal and fetal outcomes and reducing the risk of diabetes in both the mother and child. The aim of this study is to evaluate the effectiveness as well as the factors affecting glycemic control by lifestyle changes in pregnant women with GDM. *Materials and Methods*: A descriptive cross-sectional study was conducted at three hospitals in the Thai Binh Province from June 2021 to May 2022. All pregnant women at 24–28 weeks of gestation, aged 18 years or older, were enrolled. GDM was diagnosed according to the guidelines of the International Association of the Diabetes and Pregnancy Study Groups. Lifestyle changes including diet and physical exercise were carried out for two weeks. The main outcome measured was successful glycemic control according to the 2018 ADA Recommendations for the Management and Treatment of GDM. *Results*: 1035 women were included and 20.2% diagnosed with GDM. After two weeks of lifestyle change intervention, 82.6% of the pregnant women with GDM had successful glycemic control. Pregnant women aged under 35 years had a 3.2 times higher rate of gestational glycemic control than those older than 35 (aOR = 3.22, *p*-value = 0.004). Women with a pre-pregnancy BMI of less than 25 had a higher rate of gestational glycemic control than those with a BMI of over 25 (aOR = 10.84, *p*-value < 0.001). Compared to women who had all three diagnostic criteria for gestational diabetes, those with two diagnostic criteria and one criterion were 3.8 times and 3 times more likely to have successful blood sugar control (aOR = 3.78, *p*-value = 0.01 and aOR = 3.03, *p*-value = 0.03, respectively). *Conclusion*: Lifestyle changes can be an effective measure for achieving glycemic control in women with GDM. Healthcare providers should consider individualized treatment plans based on the specific needs of each patient.

## 1. Introduction

Gestational diabetes mellitus (GDM) is a type of diabetes that develops during pregnancy and affects approximately 10% of pregnant women worldwide [1]. GDM is associated with adverse maternal and fetal outcomes, including macrosomia, pre-eclampsia, preterm birth, and an increased risk of developing type 2 diabetes later in life [2]. Additionally, compared to healthy pregnant women, those with GDM had a significant decrease in sensation level, position sense and balance level compared [3]. While medical interventions such as insulin therapy and medication management are often used to manage GDM, lifestyle changes have been shown to improve glycemic control and reduce the need for medical intervention [4,5,6].

Dietary modifications are a key component of lifestyle interventions for GDM. A low-glucose diet, which emphasizes foods that have a lower impact on blood sugar levels, has been shown to improve glycemic control and reduce the need for insulin therapy in women with GDM [7]. One randomized controlled trial found that women with GDM who followed a low-glucose diet had lower fasting glucose levels and a lower incidence of macrosomia compared to those who followed a conventional high-fiber diet [8].

Physical activity is another important method of lifestyle interventions for GDM management. Exercise has been shown to improve glycemic control and insulin sensitivity in women with GDM [9]. A previous study found that regular aerobic exercises and moderate-intensity resistance training (50–55 min/session, three times/week) can be used to reduce important adverse outcomes associated with GDM [10]. The American College of Obstetricians and Gynecologists recommends at least 150 min of moderate-intensity aerobic exercise per week for women with uncomplicated pregnancies, and this recommendation can be extended to women with GDM who have been cleared for exercise by their healthcare provider [11]. Physical activity during pregnancy is safe and desirable; therefore, pregnant women should be encouraged to continue or to initiate safe physical activities and in the absence of contraindications [11].

Weight management is also an important measure of lifestyle interventions for GDM. Women who are overweight or obese before pregnancy are at a higher risk of developing GDM, and excessive gestational weight gain can further exacerbate this risk [12,13]. A previous study found that women with GDM who participated in a lifestyle intervention program that included dietary counseling, physical activity, and weight management had better glycemic control and a lower incidence of large-for-gestational-age infants compared to those who received standard care [14]. However, it is important to note that weight loss interventions during pregnancy should be carefully monitored and individualized to ensure the safety of both the mother and fetus.

Understanding the impact of lifestyle changes on glycemic control in GDM is important for improving maternal and fetal outcomes and reducing the risk of diabetes in both the mother and child. By incorporating dietary modifications, physical activity, and weight management into the management of GDM, healthcare providers can help women achieve optimal glycemic control and improve long-term maternal–fetal health outcomes.

While lifestyle interventions have shown promise in the prevention of GDM, the effectiveness of these measures on blood sugar control in pregnant women with gestational diabetes has not been clearly studied. There is still much to be learned about their effectiveness and long-term impact. Further research is needed to determine the optimal type, frequency, and intensity of lifestyle interventions for the management of GDM. Additionally, barriers to the implementation and maintenance of lifestyle changes during pregnancy need to be identified and addressed to ensure successful implementation. Therefore, we carried out this study to evaluate the effectiveness as well as the factors affecting glycemic control by lifestyle changes in pregnant women with gestational diabetes.

## 2. Materials and Methods

### 2.1. Study Design and Study Participants

This was a descriptive cross-sectional study conducted at three hospitals in Thai Binh Province, Vietnam, including Medicine University, Provincial General, and Gyneco-Obstetrics Hospital. This was a conventional study population consisting of all pregnant women who were between 24th and 28th weeks of gestation and attended clinics in the province from June 2021 to May 2022.

### 2.2. Selection and Exclusion Criteria

All pregnant women aged 18 and above at the 24th to 28th weeks of gestation coming for obstetric consultation during the study period were included. They underwent an oral glucose tolerance test. We intervened in all pregnant women diagnosed with GDM. Pregnant women with diabetes mellitus; currently suffering from diseases that affect glucose metabolism (hyperthyroidism, hypothyroidism, adrenal cortex disease, adrenal medulla disease, acute hepatitis, chronic hepatitis, using drugs that affect glucose metabolism); currently suffering from acute illnesses; having contraindications to exercise regimes; having an indication for blood glucose control using insulin; or did not agree to participate in the study or did not record enough information in the follow-up diary were excluded from this study.

### 2.3. Diagnostic Criteria

GDM was diagnosed according to the guidelines of the International Association of the Diabetes and Pregnancy Study Groups (IADPSG) using the 75 g oral glucose tolerance test with three venous blood samples [15]. The diagnostic thresholds were as follows: (1) fasting blood glucose level of 5.1–6.9 mmol/L (92–125 mg/dL); (2) 1 h blood glucose level of ≥10.0 mmol/L (180 mg/dL); and (3) 2 h blood glucose level of 8.5–11.0 mmol/L (153–199 mg/dL).

### 2.4. Lifestyle Intervention

All pregnant women with GDM without contraindications were advised on diet and exercise. They were instructed to monitor their blood glucose levels at home, perform the blood glucose testing procedure, and record the results in a monitoring notebook. The monitoring period was 2 weeks. The lifestyle intervention was carried out in three steps:

Step 1: Investigate the dietary habits of pregnant women with GDM based on a questionnaire, including the number of meals per day, the number of main and snack meals, favorite foods, and food allergies. Physical activity was investigated by asking and recording the types and duration of physical activity and exercise of patients in a day, the number of days with exercise per week.

Step 2: Prescribe nutrition and physical activity based on the individual’s diet and physical activity. The nutrition prescription is a dietary regimen for pregnant women that includes total energy, amounts of carbohydrates, fats, and proteins, and amounts of food to be consumed per day, with reference menus. Energy needs are calculated based on recommended nutritional needs for Vietnamese people [16], pre-pregnancy body mass index (BMI), current weight, weight gain during pregnancy, current gestational age, and physical activity level.

Physical activity guidance for GDM pregnant women is based on the 2018 Recommendations of the Vietnam Endocrinology–Diabetes Association [17]. Pregnant women were encouraged to engage in upper body exercises to maintain overall fitness and muscular strength. The recommended upper body exercises included walking, yoga, arm lifts, stationary cycling, swimming, and gentle aerobics for 15–30 min. Expectant mothers who have been physically active before pregnancy were generally advised to continue exercising during gestation. However, it was crucial to reduce the exercise intensity if they exercised heavily before conception. On the other hand, pregnant women with no history of exercise should gradually increase the intensity and frequency of physical activity, starting with light exercises and progressing to moderate ones. During exercise, pregnant women were advised to monitor their heart rate to ensure safe participation. The recommended heart rate limit is 140 beats per minute. Additionally, they should avoid sustaining tachycardia for more than 20 min during each exercise session. This precautionary measure aims to prevent undue stress on the cardiovascular system and minimize potential risks to both the mother and fetus [17]. A questionnaire was used to investigate physical activities during the period of intervention, including the type of exercise, number of exercises per day, duration, and time of each exercise session [17].

Step 3: Monitor pregnant women for nutrition and physical activity control during treatment and record their daily diet as well as physical activity. The timing of blood glucose testing is based on the 2018 ADA Recommendations for the Management and Treatment of GDM [18]. Within two weeks of intervention, pregnant women had to perform capillary blood glucose testing three times per day using a glucometer and test strip at the following times: before meals, 1 h after meals, and 2 h after meals. Depending on the conditions of the pregnant woman, blood glucose testing can be performed after one of the three main meals: breakfast, lunch, or dinner. All the results of blood glucose test are recorded in the blood glucose monitoring notebook.

### 2.5. Evaluation of Intervention Effectiveness

The effectiveness of lifestyle interventions was evaluated based on the results of a capillary blood glucose test. According to the ADA and National Guidelines for Prevention and Control of GDM [16,17,19], the blood glucose target for pregnant women with GDM was as follows: (1) before meals: ≤5.3 mmol/L (95 mg/dL), (2) 1 h after meals: ≤7.8 mmol/L (140 mg/dL), and (3) 2 h after meals: ≤6.7 mmol/L (120 mg/dL).

The intervention results are evaluated after 2 weeks of intervention according to the 2018 Recommendations of the Vietnam Endocrinology–Diabetes Association [17]. The results were considerably successful when 80% or more of the samples of capillary blood glucose tests reached the blood glucose control target for pregnant women with GDM. Pregnant women who achieved treatment results are instructed to continue to change their lifestyle and monitor their capillary blood glucose levels until delivery. Pregnant women whose blood glucose levels did not reach the target were advised and prescribed insulin for the treatment.

**Figure 1:**
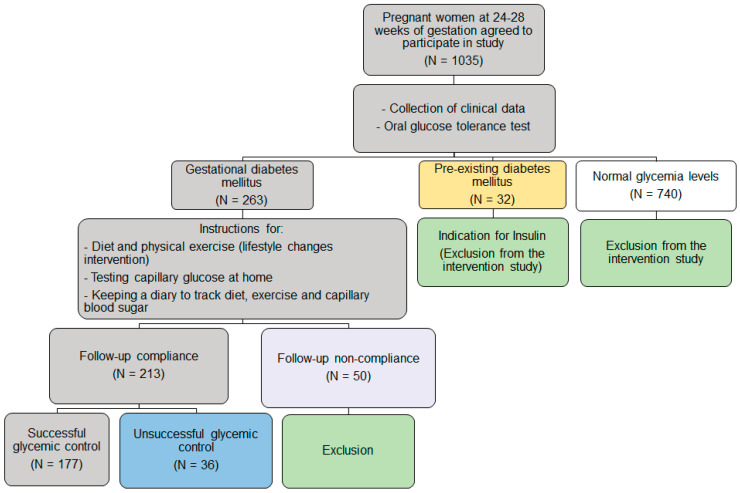
Study design.

Figure 1 shows the study design.

### 2.6. Data Analysis

The data were entered using Epidata 3.1 software. The dataset was double-checked for errors. Stata v.17.0 software was used to analyze the data. The success rate of controlling blood glucose was used to evaluate the effectiveness of the intervention. A Chi-square test was used to evaluate the difference in proportions. The main outcome measured was successful glycemic control. The association between the main outcome and independent factors such as maternal age, education level, occupation, chronic diseases, BMI, diabetes mellitus in family members, history of pregnancy, pregnancy method, and the number of criteria for GDM diagnosis was firstly evaluated using univariate analysis. Variables with a *p*-value < 0.25 in the univariate analysis were imported into the multivariate analysis using logistic regression [20]. The results were presented as odds ratio (OR) and 95% confidence interval (95%CI). A *p*-value < 0.05 was considered statistically significant.

## 3. Results

### 3.1. Characteristics of Participants

**Figure 2:**
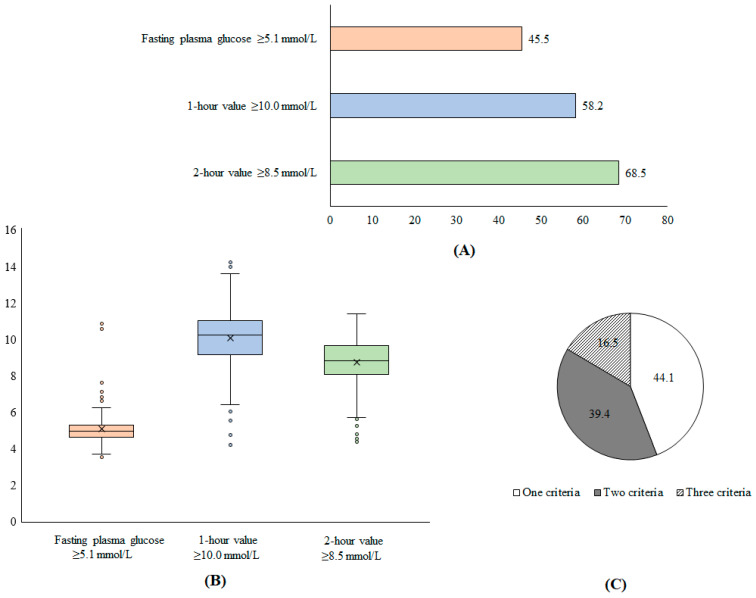
Proportion of patients with GDM according to diagnostic criteria (**A**), distribution of glycemic levels (**B**), and proportion of patients with number of diagnostic criteria for GDM (**C**).

During the inclusion period, a total of 1035 pregnant women were enrolled and agreed to participate in our study, of whom 263 (20.2%) met the criteria for GDM. Figure 2 shows the distribution of glycemia among women with GDM. A total of 45.5% patients had fasting plasma glucose ≥ 5.1 mmol/L while 58.2%, and 68.5% of participants had 1 h value ≥ 10.0 mmol/L and 2 h ≥ 8.5 mmol/L, respectively. Furthermore, 44.1% of patients met one of the criteria for GDM diagnosis, while 39.4% and 16.5% met two and three criteria, respectively (Figure 2).

### 3.2. Effectiveness of Lifestyle Changes in Glycemic Control among Pregnant with GDM

After lifestyle changes, glycemia was successfully controlled in 176/263 patients (82.6%). Table 1 shows the characteristics of both the successful glycemic control group and the unsuccessful glycemic control group.

A total of 30.5% of patients were aged ≥35 years. Chronic diseases occurred in 8.9% of women with GDM. A total of 6.6% of patients had a pre-pregnancy BMI ≥ 25.

The factors affecting the effectiveness of lifestyle changes in glycemic control among pregnant women with GDM are presented in Table 2.

The multivariate analysis showed that pregnant women aged under 35 years had a 3.2 times higher rate of gestational glycemic control compared to those older than 35 (aOR = 3.22, 95%CI = [1.45–7.14], *p*-value = 0.004). Women with a pre-pregnancy BMI of less than 25 had a higher rate (11 times) of gestational glycemic control than those with a BMI over 25 (aOR = 10.84, 95%CI = [3.08–38.18], *p*-value <0.001). The number of diagnostic criteria for gestational diabetes was inversely proportional to the ability to control blood sugar. Specifically, compared to women who had all three diagnostic criteria for gestational diabetes, those with two diagnostic criteria were 3.8 times more likely to achieve successful blood sugar control (aOR = 3.78, 95%CI = [1.35–10.55], *p*-value = 0.01). Similarly, women with only one diagnostic criterion of gestational diabetes were three times more likely to have successful blood sugar control (aOR = 3.03, 95%CI = [1.11–8.25], *p*-value = 0.03).

## 4. Discussion

The results of our study suggest that lifestyle changes can be an effective measure for glycemia control in women with GDM. Of the 263 pregnant women who met the criteria for gestational diabetes, 82.6% were able to achieve successful blood sugar control through lifestyle changes. This is an important finding, as gestational diabetes can lead to adverse outcomes for both the mother and the baby, including an increased risk of preterm delivery, macrosomia, and neonatal hypoglycemia. This finding is consistent with the previous studies, where 70–85% of pregnant women with gestational diabetes could control gestational diabetes simply by changing their lifestyle [21]. According to a randomized controlled trial on 1220 women with GDM, Gadgil et al. showed that even a small improvement in diet quality may be beneficial for the achievement of improved glycemic control [22]. Although the rates of achieving the glycemic goal varied between studies, depending on the sample size of studies, they were all relatively high. This suggests that non-pharmacological interventions such as diet and physical activity are important and useful as the primary treatment for glycemic control in most women with GDM.

Regular physical exercise can be a beneficial approach to optimize glucose regulation during pregnancy, leading to reduced blood glucose levels and improved insulin sensitivity [8,23,24,25]. This, in turn, helps lessen the burden on the compensating β-cells. Exercise achieves a decrease in blood glucose concentration through two distinct pathways: the contraction-mediated pathway and the insulin-stimulated pathway. The physiological mechanisms responsible for increased insulin sensitivity include a rise in the number of insulin-sensitive glucose transporters (GLUT-4), improved response of GLUT-4 to insulin, and increased glycogen synthase activity within skeletal muscles. This combination of factors works together to lower capillary glucose concentrations. Since skeletal muscle is the primary site for insulin-stimulated glucose uptake, any intervention aimed at enhancing glucose uptake in this tissue will improve overall insulin sensitivity in the body. The metabolic advantages of exercise during gestational diabetes (GDM) are believed to stem from changes affecting pathways that influence insulin sensitivity, adipokines, and reduction–oxidation reactions [8]. A comprehensive report published by Health and Human Services, USA, in 2015 indicated that physical activity significantly improved abnormal glucose tolerance primarily caused by insulin resistance, rather than when it was due to deficient amounts of circulating insulin [23]. Furthermore, exercise has been shown to reduce intra-abdominal fat, a known risk factor for insulin resistance. Several studies have found an inverse association between physical activity and intra-abdominal fat distribution, leading to reduced body fat stores [24].

Research on interventional studies has indicated that high carbohydrate and high monounsaturated fat diets can lead to improved insulin sensitivity [24]. For diabetic patients with dyslipidemia, implementing dietary measures aimed at glucose disposal is often the first line of intervention for control. Several interventional studies have highlighted the importance of nutrition therapy and lifestyle changes as the primary treatment for dyslipidemia. In managing diabetes and its associated complications, maintaining metabolic control is essential. Of particular concern for people with diabetes is the impact of carbohydrate intake on postprandial glucose levels, making it a significant factor in glycemic management. Additionally, an individual’s food choices and energy balance directly influence body weight, blood pressure, and lipid levels. To achieve health goals, it is crucial for healthcare professionals to collaborate with their patients, offering personalized nutrition interventions and ongoing support to facilitate positive changes [24].

Our study also identified several factors that may influence the success of glycemic control in women with GDM. Younger age was associated with higher rates of successful glycemic control, with pregnant women under the age of 35 having a 3.2 times higher rate of glycemic control than those over the age of 35. This may be due to younger women being more receptive to lifestyle changes, or having fewer underlying health conditions that could interfere with glycemic control. Maternal age at pregnancy is a factor closely related to gestational diabetes. In a study evaluating the relationship between maternal age and serum glucose levels during pregnancy in 424 pregnant women between 24 and 28 weeks of gestation, an increase in serum glucose and the incidence of diabetes is significantly higher with increasing maternal age [25]. In a retrospective case–control study conducted in northern California among 26,397 women, age was significantly associated with risk for GDM [26]. In addition, advanced maternal age may also be at risk of many adverse events during pregnancy and for the fetus [27].

Another important factor that influences the management of GDM was pre-pregnancy BMI, with women who had a BMI under 25 having a much higher rate of glycemic control than those with a BMI over 25. Several studies have shown that high pre-pregnancy BMI was a vital factor in GDM [6,25,28,29,30]. But the impact of pre-pregnancy BMI on the effectiveness of lifestyle changes in controlling glycemia in women with GDM is not well studied. Our finding highlights the importance of pre-pregnancy counseling and weight management in reducing the risk of GDM and its associated complications.

Interestingly, our study found that the number of diagnostic criteria for gestational diabetes was inversely proportional to the ability to control blood glucose among pregnant women with GDM. Specifically, women who had all three diagnostic criteria for GDM were less likely to achieve successful glycemic control than those with only one or two diagnostic criteria. Indeed, GDM is a condition that occurs when the pancreas does not produce enough insulin to handle the rise in blood sugar, due to the increase in placental hormone secretion during pregnancy. Our results could be explained by the fact that women with more diagnostic criteria had more severe or advanced GDM, making it less effective to control blood glucose with lifestyle changes. Our findings highlight the importance of early screening for gestational diabetes for prompt intervention.

It is important to note that while lifestyle changes can be effective in controlling blood glucose levels in women with GDM, these changes may not be sufficient for all women. In cases where glycemic control cannot be achieved through lifestyle changes alone, medication or insulin therapy may be necessary. Therefore, it is important for healthcare providers to individualize treatment plans based on the specific needs and circumstances of each patient.

There are several limitations to our study that should be noted. First, our study only included pregnant women who agreed to participate, which may have introduced selection bias. Additionally, our study was conducted in a single center, which may limit the generalizability of our findings to other populations. Furthermore, this was a conventional study and the levels of the influencing parameters were not investigated. This might lead to confusion in the conclusion. Finally, lifestyle changes, physical activity, and diet changes are the appropriate initial proposal for pregnant women with newly diagnosed GDM, but the standard for the monitoring of GDM patients is more intensive. The daily monitoring of the symptoms of ketosis is extremely important in pregnant women with GDM because the process of gluconeogenesis (and finally ketosis) could be dangerous for pregnant women and especially their babies. Moreover, for the appropriate monitoring of GDM patients, the serial ultrasound monitoring of the fetus is also needed to evaluate the development of pregnancy (biometry of the fetus-head and abdominal circumferences, macrosomia or fetal growth restriction, and amniotic fluid volume). However, we did not evaluate the daily urine ketone levels of pregnant women and did not monitor fetal growth through ultrasound, nor the outcomes of mothers and fetuses after delivery.

## 5. Conclusions

Our study provides evidence that lifestyle changes can be an effective measure of glycemic control in women with GDM. Younger age, lower pre-pregnancy BMI, and fewer diagnostic criteria for gestational diabetes were associated with higher rates of successful glycemic control. Healthcare providers should consider individualized treatment plans based on the specific needs of each patient, and further research is needed to confirm these findings in larger and more diverse populations.

## Figures and Tables

**Table 1:** Characteristics of included patients with GDM.

Characteristics		Unsuccessful Glycemic Control Group		Successful Glycemic Control Group		Total		*p*-Value
		*n*	%	*n*	%	*n*	%	
Age	<35	16	43.2	132	75.0	148	69.5	<0.0001
	≥35	21	56.8	44	25.0	65	30.5	
Education level	≤High school	15	40.5	61	34.7	76	35.7	0.50
	>High school	22	59.5	115	65.3	137	64.3	
Occupation	Housewife, farmer, small business	13	35.1	48	27.3	61	28.6	0.56
	Worker	10	27.0	41	23.3	51	23.9	
	State officials	6	16.2	24	13.6	30	14.1	
	Private officer	6	16.2	52	29.5	58	27.2	
	Others	2	5.4	11	6.3	13	6.1	
Chronic diseases	No	33	89.2	161	91.5	194	91.1	0.66
	Yes	4	10.8	15	8.5	19	8.9	
Pre-pregnancy body mass index	<25	28	75.7	171	97.2	199	93.4	<0.0001
	≥25	9	24.3	5	2.8	14	6.6	
Diabetes mellitus in family	No	36	97.3	172	97.7	208	97.7	0.88
	Yes	1	2.7	4	2.3	5	2.3	
History of pregnant	First	11	29.7	72	40.9	83	39.0	0.21
	≥Second	26	70.3	104	59.1	130	61.0	
Pregnancy method	Nature	26	70.3	155	88.1	181	85.0	0.01
	IVF	11	29.7	21	11.9	32	15.0	
Number of criteria for GDM diagnosis	1	14	37.8	80	45.5	94	44.1	0.02
	2	11	29.7	73	41.5	84	39.4	
	3	12	32.4	23	13.1	35	16.4	

**Table 2:** Factors affecting the effectiveness of lifestyle changes in glycemic control among pregnant women with GDM.

Characteristics		Univariate Analysis			Multivariate Analysis *		
		OR	95% CI	*p*	OR	95% CI	*p*
Age	≥35	reference			reference		
	<35	3.94	1.89–8.20	<0.001	3.22	1.45–7.14	0.004
Education level	>High school	reference					
	≤High school	0.78	0.38–1.61	0.50			
Occupation	Housewife, farmer, small business	reference			reference		
	Worker	1.11	0.44–2.80	0.82	0.84	0.28–2.50	0.76
	State officials	1.08	0.37–3.20	0.89	0.87	0.24–3.11	0.83
	Private officer	2.35	0.83–6.67	0.11	1.40	0.43–4.52	0.58
	Others	1.49	0.29–7.58	0.63	1.04	0.17–6.24	0.96
Chronic diseases	Yes						
	No	1.30	0.40–4.17	0.66			
Pre-pregnancy body mass index	≥25	reference			reference		
	<25	10.99	3.43–35.20	<0.001	10.84	3.08–38.18	<0.001
Diabetes mellitus in family	Yes	reference					
	No	1.19	0.13–11.00	0.88			
History of pregnant	≥Second	reference			reference		
	First	0.61	0.28–1.32	0.21	1.17	0.46–2.96	0.74
Pregnancy method	IVF	reference			reference		
	Nature	3.12	1.35–7.23	0.01	2.21	0.88–5.56	0.09
Number of criteria for GD diagnosis	3	reference			reference		
	2	3.46	1.35–8.89	0.01	3.78	1.35–10.55	0.01
	1	2.98	1.21–7.33	0.02	3.03	1.11–8.25	0.03

* Only variables with a *p*-value < 0.25 in the univariate analysis were introduced into the multivariate analysis.

## Data Availability

The data presented in this study are available on request from the corresponding author [V.T.H] upon reasonable request.
